# Global transcriptional response of *Saccharomyces cerevisiae *to the deletion of *SDH3*

**DOI:** 10.1186/1752-0509-3-17

**Published:** 2009-02-06

**Authors:** Donatella Cimini, Kiran R Patil, Chiara Schiraldi, Jens Nielsen

**Affiliations:** 1 Second University of Naples, Department of Experimental Medicine, Naples, Italy; 2 Center for Microbial Biotechnology, Department of Systems Biology, Technical University of Denmark, Building 223, DK-2800 Kgs. Lyngby, Denmark; 3 Department of Chemical and Biological Engineering, Chalmers University of Technology, Gothenburg, SE-412 96, Sweden

## Abstract

**Background:**

Mitochondrial respiration is an important and widely conserved cellular function in eukaryotic cells. The succinate dehydrogenase complex (Sdhp) plays an important role in respiration as it connects the mitochondrial respiratory chain to the tricarboxylic acid (TCA) cycle where it catalyzes the oxidation of succinate to fumarate. Cellular response to the Sdhp dysfunction (i.e. impaired respiration) thus has important implications not only for biotechnological applications but also for understanding cellular physiology underlying metabolic diseases such as diabetes. We therefore explored the physiological and transcriptional response of *Saccharomyces cerevisiae *to the deletion of *SDH3*, that codes for an essential subunit of the Sdhp.

**Results:**

Although the Sdhp has no direct role in transcriptional regulation and the flux through the corresponding reaction under the studied conditions is very low, deletion of *SDH3 *resulted in significant changes in the expression of several genes involved in various cellular processes ranging from metabolism to the cell-cycle. By using various bioinformatics tools we explored the organization of these transcriptional changes in the metabolic and other cellular functional interaction networks.

**Conclusion:**

Our results show that the transcriptional regulatory response resulting from the impaired respiratory function is linked to several different parts of the metabolism, including fatty acid and sterol metabolism.

## Background

Mitochondrial respiration plays a central role in energy metabolism in eukaryotic cells. It is involved in generating energy, primarily in the form of ATP, upon oxidation of different carbon sources such as ethanol, pyruvate and diverse organic acids. This functionality is conserved across all eukaryotic cells. Consequently, metabolic and regulatory mechanisms governing the mitochondrial energy generation have many implications for the functioning of the cell as a whole. A primary component of mitochondrial metabolism is the TCA cycle which apart from producing NADH needed in the oxidative phosphorylation also supplies precursors for biomass synthesis, e.g. 2-oxoglutarate and oxaloacetate. The coupling between metabolism and oxidative phosphorylation is also reflected in the tight transcriptional regulation of the TCA cycle, for example, as observed in *Saccharomyces cerevisiae *during the diauxic shift between fermentative and oxidative metabolism. The TCA cycle is also known to be transcriptionally regulated in response to oxygen and carbon substrate concentrations. The succinate dehydrogenase complex (Sdhp) serves as a link between the TCA cycle and electron transport chain. Specifically, FADH_2 _produced during oxidation of succinate to fumarate acts as an electron donor for ubiquinone. Thus, the flux through the succinate dehydrogenase reaction is directly coupled with the respiratory capacity of the cell. The respiratory chain itself is believed to be transcriptionally regulated by oxygen and heme concentrations, and several other not well characterized mechanisms, e.g. in response to osmotic stress [1].

Defects in the respiratory chain and related energy metabolism have been shown to be key factors in inducing several human diseases, including diabetes, obesity and certain types of cancers [2-6]. Consequently, respiratory metabolism has been a major focus for studying varying diseases, and there is much interest in using appropriate eukaryotic model systems that can be used for functional studies. The yeast *S. cerevisiae *has been widely used as a model eukaryotic organism as many cellular processes are conserved to humans, and comparatively large amounts of genomic, metabolomic, and proteomic data is readily available. Furthermore, *S. cerevisiae *is industrially used for producing several commodity and high added-value compounds, such as bio-ethanol and insulin. Consequently, there is much interest in developing rational design strategies for metabolically engineering yeast to improve the production of desired compounds. Previous studies have suggested the TCA cycle and energy metabolism as primary targets for certain metabolic engineering problems [7,8].

The Sdhp is a tetramer consisting of two soluble subunits responsible for the dehydrogenase activity, and two hydrophobic subunits that anchor the catalytic subunits to the mitochondrial inner membrane. The *SDH3 *gene in *S. cerevisiae *codes for the cytochrome b component of the complex, and as shown previously [9], disruption of the gene leads to a severe growth defect on non-fermentable carbon sources demonstrating its major role in the complex assembly and function. In light of the importance of Sdh3 for improved understanding of human diseases and metabolic engineering applications, we characterized the physiology of *sdh3*Δ mutant and performed genome wide transcription analysis. The cultivation type was chosen to be batch fermentation with glucose as the sole carbon source so as to have glucose-repressed conditions.

## Methods

### *SDH3 *knockout strain construction

The reference *S. cerevisiae *strain CENPK 113–5D (*Mat a MAL2–8C SUC2 URA3–52*) was used for the construction of the *sdh3*Δ knockout strain through the cloning-free PCR-based allele replacement method previously described [10]. The upstream *SDH3 *fragment was amplified by PCR from genomic DNA using the primers SDH3_Up_Fw (sequence 5'-CCGAAATATGGTAAGAGAAAATG-3') and SDH3_Up_Rv (sequence 5'-GCAGGGATGCGGCCGCTGACGACATCGTTTATTATTCTTAGAGC-3'). Similarly, the downstream *SDH3 *fragment was amplified using the primers SDH3_Dw_Fw (sequence 5'CCGCTGCTAGGCGCGCCGTGCTTTATGATTCTTTAAGGCGACGC-3') and SDH3_Dw_Rv (sequence 5'-GTAATCTGTTATCGATAATCTGCC-3'). Lithium acetate transformation was employed [11]. As described previously [10], *URA3 *from *Kluyveromyces lactis *was used as the selection marker in the transformation process. With this approach, transformants can easily be selected on media lacking uracil, and direct-repeat recombinants can be identified by counter-selecting on media containing 5-Fluoroorotic Acid. The knockout was confirmed by restriction analysis and sequencing (MWG Biotech AG). Yeast extract-peptone-dextrose (YPD) medium, synthetic complete (SC) medium, and SC lacking specific amino acids were prepared as described previously [12].

### Growth conditions

All cultures were grown in minimal media prepared according to Verduyn *et al*. [13] supplemented with 150 mg/liter of uracil to complement auxotrophy for the deletion mutant strain. Before each experiment, cells from 15% glycerol stock preparations were plated on YPD medium (10 g/liter yeast extract, 20 g/liter of peptone, 20 g/liter glucose) and incubated at 30°C for 24–48 h. A single colony was then used to inoculate pre-cultures.

### Batch fermentation

Batch experiments for physiological characterization of the strains were carried out in a Braun Biostat-B reactor with a working volume of 1.5 liter and 10 g/liter initial glucose concentration. A constant pH of 5 was maintained *via *automated addition of 2 M NaOH. Aerobic conditions were established by supplying air at the rate of 1.5 liter/min whereas sparging with pure N_2 _established anaerobic conditions. The concentration of CO_2 _and O_2 _in the exhaust gas was determined by the use of a Brüel & Kjær acoustic gas analyzer (Brüel & Kjær, Denmark). For anaerobic cultivation the medium was supplemented with ethanol-dissolved ergosterol and Tween 80 (Sigma-Aldrich) to a final concentration of 0.01 g/liter and 0.42 g/liter, respectively.

Five liter in-house-manufactured bioreactors with 4 liter working volume were used to carry out aerobic batch cultivations for microarray experiments. The initial glucose concentration was increased to 40 g/liter and therefore the concentration of all medium components was accordingly adjusted. The cultivations were carried out in at least triplicate.

### Chemostat cultivation

C-limited chemostat experiments were also performed in Braun Biostat B with the same process variables as described above for the aerobic batch, at a dilution rate of 0.027 h^-1 ^for the *sdh3*Δ strain and 0.05 h^-1 ^for the wild type strain (WT). Steady state samples for extracellular metabolite analysis were collected after 3 volume changes.

### Inhibitor experiments

Shake flask experiments were also performed to analyse the effects of 3 specific inhibitors of the respiratory chain. The *SDH3 *mutant and the reference strain were grown in minimal media and treated with antimycin (1 *μ*g/ml), oligomycin (3 *μ*g/ml) or carbonyl cyanide m-chlorophenylhydrazone (CCCP) (4.1 *μ*g/ml) (Sigma-Aldrich) during the early exponential phase.

### Sampling

During the course of all cultivations 5 ml samples were withdrawn from the bioreactor at regular time intervals for the determination of dry cell weight and extracellular metabolites. Glucose, ethanol, pyruvate, succinate, acetate, fumarate, citrate, oxaloacetate and malate were quantified by HPLC using a Biorad HPX-87H column.

### RNA isolation and microarray analysis

Duplicate samples for microarray analysis were collected during the aerobic batch experiments with the *sdh3*Δ strain when the glucose concentration had reached 20 ± 2 g/liter. Culture aliquots of 20 ml were transferred to falcon tubes already filled with 25 ml of crushed ice and immediately centrifuged at 4000 rpm for 5 minutes. The pellets were rapidly frozen in liquid nitrogen and stored at -80°C until further use. Total RNA was extracted by using the Fast Pro Red Kit (Obiogene, USA) according to the manufacture's instructions with minor modifications. cRNA was synthesized as described in the Affymetrix GeneChip Expression Analysis Manual, after which 15 *μ*g were hybridized to Yeast Genome S98 oligonucleotide arrays (Affymetrix). Microarrays were scanned in Agilent Gene Scanner (Affymetrix), following which raw data processing was performed with the Microarray Suite software, version 5, with a global scaling factor for target intensity equal to 500. Average difference values, representing the absolute hybridization intensities were then calculated for each probe set. The microarray data was first normalized by using dChip software suit (dChip, version 1.3, Wong Lab, Harvard School of Public Health and Dana-Farber Cancer Institute, Boston MA, USA). Expression levels of all 9335 probes sets were calculated with the Perfect Match model using dChip v1.3. From the 9335 probe sets in the array, the expression level of 6079 annotated unique Open Reading Frames (ORFs) from *Saccharomyces *Genome Database were extracted. Significance of expression change was calculated in terms of *p*-values for all the genes by using Student's t-test.

Microarray data were deposited in the Gene Expression Omnibus (GEO) database  under accession number GSE13924.

### Reporter algorithm

In order to identify key proteins involved in the cellular response to *SDH3 *deletion, we used a systems approach where bio-molecular interaction networks are used as data integration scaffolds [14,15]. Three different interaction networks were used in this study, *viz*. metabolic network [16], regulators/Transcription Factors (TFs)-'regulated genes' network [15], and 'Gene Ontology' (GO)-'associated genes network' [15]. All three networks can be conceptually seen as a graph where the feature nodes (metabolites, GOs or TFs) are connected with the corresponding functionally linked gene nodes. The *p*-values for the individual genes were then mapped on each of these networks and the features with significant transcriptional response in their neighbour genes were identified as reporter features, i.e., reporter metabolites, reporter GOs and reporter TFs. The scoring method used for estimating the significance score for the features is summarized in the following three equations [15]:

(1) *z*_*gene i *_= *cdf*^-1 ^(1- *p*_*gene i*_)

(2) $$z_{feature\;j}=\frac{1}{N}\sum_{k=1}^{N}z_{gene\;k}$$

(3) $$z_{feature\;j}^{corrected}=\frac{\left(z_{feature\;j}-\mu_{N}\right)}{\sigma_{N}}$$

First, p-value for each gene *i *in the network, *p*_*genei*_, was converted into a z-score by using the inverse normal cumulative distribution function (*cdf*^-1^). The resulting z-scores approximately follow a standard normal distribution. The score for each feature *j *(*z*_*featurej*_) was then calculated as the average of the z-scores of its neighbour nodes (equation 2). To assess the significance of each *z*_*featurej*_, it was corrected for the background distribution of z scores, by subtracting the mean (*μ*_*N*_) and then dividing by the standard deviation (*σ*_*N*_) of 10000 random aggregates of size *N *selected from the same data; *N *being the number of neighbours for the feature under consideration. The resulting reporter features can be seen as nodes in the bio-molecular interaction networks around which there are substantial transcriptional changes occurring. These nodes are a metabolite interacting with different enzymes, a transcription factor regulating the transcription of several genes or a common annotation for a group of genes, e.g. GO annotation.

The reporter scores calculated in this study do not require *a priori *selection of a p-value cut-off at the individual gene level. It may be also of interest to observe the enrichment in the number of significantly changed genes around a particular feature, whereas the cut-off level at the individual gene level is chosen *a priori*. The biological meaning of such scoring method is discussed by Oliveira *et al *[15]. The software for calculating such enrichment scores is freely available at: .

### Promoter analysis

A bioinformatics analysis was performed by using Regulatory Sequence Analysis web-tools [17] in order to identify potential regulatory elements in the promoter region (800 bp region upstream of each start codon) of the genes that showed a significant change in expression following the *SDH3 *deletion.

## Results

### Physiological characterization

In order to study the physiological consequences of *SDH3 *deletion, a comparison between the reference and mutant strain was performed under well-controlled aerobic and anaerobic batch fermentations in minimal medium with glucose as the sole carbon source. The *sdh3*Δ mutant showed a lower maximum specific growth rate (0.25 h^-1^) in aerobic growth conditions as compared to the reference strain (0.31 h^-1^). Furthermore, the mutant was incapable of utilizing ethanol as a sole carbon source, as also been reported previously [9]. A similar phenotypic difference was observed in anaerobic batch cultivations, where the maximum specific growth rates were 0.27 h^-1 ^and 0.34 h^-1 ^for the mutant and the reference strain, respectively. Since, under both aerobic and anaerobic conditions the cells are mainly having fermentative metabolism, the lower specific growth rate observed in both conditions are likely to be due to the same mechanisms.

The yield coefficients of biomass and secreted metabolites with respect to consumed glucose are listed in Table 1. We observed a slight increase in succinate production in the mutant strain; yield of 0.0033 g/g-glucose was obtained in anaerobic growth conditions whereas no (or undetectable amounts) succinate was produced by the reference strain. The physiology of the mutant was also characterized in aerobic, glucose limited chemostat cultivations at low dilution rates. The results indicate that the respiratory metabolism is non-functional in the mutant, as pronounced fermentative metabolism was observed at dilution rates as low as 0.027 h^-1^.

**Table 1:** Physiological characterization of the reference and *sdh3*Δ strains in aerobic and anaerobic batch cultivations

**Strain**	**Condition**	***μ*_max_**	**Y_sx_**	**Y_ssuc_**	**Y_se_**	**Y_sac_**	**Y_sg_**	**Y_spyr_**
*sdh3*Δ	Aerobic	0.25 ± 0.03	0.083 ± 0.003	0.0027 ± 0.0001	0.35 ± 0.002	0.0062 ± 5.7·10^-5^	0.108 ± 0.004	0.0061 ± 0.0003
*sdh3*Δ	Anaerobic	0.27	0.086	0.0033	0.36	0.0065	0.097	0.0041
WT	Aerobic	0.31 ± 0.04	0.10 ± 0.01	TA	0.34 ± 0.01	0.0042 ± 0.0008	0.073 ± 0.010	TA
WT	Anaerobic	0.34	0.10	0	0.35	0.0050	0.106	0.0027

The yield coefficients for biomass, ethanol, glycerol, pyruvate and acetate were determined in the exponential growth phase from linear regression of their concentration as a function of the residual glucose concentration. Succinate yield was calculated at a fermentation end-point. Y_sx_: grams of biomass per g of glucose consumed. Y_ssuc_: grams of succinate per g of glucose consumed. Y_se_: grams of ethanol per g of glucose consumed. Y_sac_: grams of acetate per g of glucose consumed. Y_sg_: grams of glycerol per g of glucose consumed. Y_spyr_: grams of pyruvate per g of glucose consumed. *μ*_max _: maximum specific growth rate in h^-1^. TA: trace amount.

### Inhibition of respiration

Shake flask experiments were performed on minimal media to study the effects of three different oxidative phosphorylation inhibitors (antimycin, oligomycin and CCCP) on the *SDH3 *mutant. Antimycin inhibits the electron transfer and thereby prevents the oxidation of both NADH and succinate. Oligomycin specifically binds to the F_0 _subunit of the ATP-synthase complex inhibiting ATP synthesis and ATP-synthesis associated respiration. The last inhibitor used, CCCP, is an uncoupling agent that allows free movement of protons across the mitochondrial inner membrane causing a collapse in the H^+ ^gradient that is required for ATP synthesis by the F_0_F_1_-ATPase. The addition of oligomycin and antimycin had no effect on the specific growth rate and biomass production in the *sdh3*Δ strain while they considerably reduced the final biomass yield in the reference strain and impaired growth on ethanol. The major inhibitory effects in both strains were observed after treatment with CCCP which resulted in reduced specific growth rate and biomass yield in the reference strain, while it completely inhibited growth of *sdh3*Δ.

### Global overview of transcriptional response to *SDH3 *deletion

To understand the cellular processes denominating the observed physiological differences in aerobic batch cultivations we performed a genome-scale transcriptional analysis of the *sdh3*Δ strain (see methods). We used the previously published data for the reference strain [18] for comparative analysis. Approximately 25% of the yeast genes showed significant changes in their expression levels (*p*-value ≤ 0.05), indicating a global transcriptional response. After applying a correction for multiple testing by using q-test [19], 307 genes were deemed as significantly changed. We also applied rank-product test [20] in order to identify the genes that were significantly up/down regulated, yielding additional 127 genes (additional file 1). The distribution of these 434 genes in different functional categories as defined by the MIPS database  is shown in Table 2. The next largest group to the 'unclassified proteins' is the group of genes belonging to RNA synthesis and processing. Moreover, several genes belonging to intracellular protein and mRNA transport (including vacuolar and endoplasmic reticulum related), carbon metabolism, cell cycle, cellular response and sensing, ribosomal biogenesis and DNA metabolism have significantly changed expression levels in the *sdh3*Δ mutant (Table 2). In general, the majority of the genes are down-regulated in each category, indicating a global repressive effect of the *SDH3 *deletion. To investigate on the possibility that the slower growth rate of the mutant may lead to decreased expression of several genes, we used the gene expression data of *S. cerevisiae *grown at several different specific growth rates in chemostat cultivations [21] to compare against the changes observed in the *sdh3*Δ mutant. It should be noted that due to the different cultivation conditions (i.e. batch *vs*. chemostat) it is not possible to draw direct conclusions on the growth rate effects in this fashion. Moreover, the effects of growth rate on gene expression are inherently linked with the metabolic state and cell cycle and thus the problem of differentiating between growth rate and perturbation effects is complex [22,23]. Nevertheless, we decided to use a simple extrapolative analysis by using the data from Regenberg *et al *[21]. Following linear fitting of expression values of each gene with growth rate, we checked whether the direction and magnitude of expression changes in the *sdh3*Δ mutant concur with those predicted by this linear model. Based on this analysis we postulate that most of the observed changes can not be directly and solely attributed to the difference in growth rate.

**Table 2:** Affected genes sorted by MIPS categories

**MIPS functional category**	**Number of genes**	**MIPS functional category**	**Number of genes**
Unclassified proteins	124 (26, 98)	DNA processing	15 (4, 11)
RNA synthesis	41 (7, 34)	Protein degradation	15 (4, 11)
Transport routes	41 (11, 30)	Ribosome biogenesis	15 (7, 8)
Cell cycle	27 (4, 23)	Nucleotide metabolism	14 (4, 10)
C-compound and carbohydrate metabolism	25 (10, 15)	Protein targeting, sorting and translocation	13 (1, 12)
RNA processing	25 (3, 22)	Cell growth/Morphogenesis	12 (3, 9)
Fungal/microrganismic cell type differentiation	23 (2, 21)	Respiration	12 (1, 11)
Cellular sensing and response	20 (1, 19)	Lipid, fatty acid and isoprenoid metabolism	11 (4, 7)
Transported compounds (substrates)	17 (8, 9)	Stress response	11 (3, 8)
Protein modification	16 (7, 9)	Detoxification	9 (4, 5)
Mitochondrion	9 (4, 5)	Ionic homeostasis	9 (3, 6)

Distribution of genes with significantly changed expression levels in the *SDH3 *deletion mutant classified in different functional categories as defined by the MIPS database. Number in parentheses show up and down regulated genes, respectively.

Although the classification of differentially expressed genes according to the MIPS functional categories provides a birds-eye view of the transcriptional response, we further investigated whether there are certain biological processes, enzyme functions or cellular locations that responded more significantly than the rest. For this purpose, we integrated the Gene Ontology network with transcription data in order to obtain reporter-Gene Ontology [15] terms. This integration algorithm uses the data from all genes without *a priori *selection of significant genes, and is capable of identifying weak but biologically significant changes [15]. Thus, reporter-GOs indicate the key biological processes/functions/cellular components that collectively respond significantly to the perturbation (for the complete list of reporter GOs see additional file 2). Reporter-GO terms for the *sdh3*Δ mutant (Table 3) clearly reflect the large transcriptional changes in nucleolus related genes (148 genes), indicating altered ribosome synthesis and transport due to *SDH3 *deletion. These changes also appear to be extended to other related processes such as ribosomal assembly and maintenance, nuclear rRNA and mRNA processing and protein localization. Appearance of "nucleus" (1071 genes) as one of the top-scoring reporter-GO terms shows that many of the nuclear genes are responding to the changes induced by defective respiration. This set also includes genes related to meiosis, DNA replication initiation, and DNA repair. These changes might be one of the reasons for reduced growth rate of the deletion mutant, particularly as they are also found to being effected upon changing growth rate in chemostat cultures [21].

**Table 3:** Reporter-Gene Ontology terms for the *sdh3*Δ mutant

**GO term**	**Number of associated genes**	**Reporter *p*-value**
nucleolus (C)	148	7.25E-10
late endosome to vacuole transport (P)	16	1.38E-04
processing of 20S pre-rRNA (P)	46	1.80E-04
small nucleolar ribonucleoprotein complex (C)	55	8.71E-04
35S primary transcript processing (P)	64	1.09E-03
rRNA processing (P)	58	1.20E-03
spliceosome complex (C)	29	1.52E-03
ubiquitin-dependent protein catabolism (P)	62	1.53E-03
transcription regulator activity (F)	20	1.63E-03
barrier septum formation (P)	1	1.64E-03
asymmetric protein localization (P)	1	1.64E-03
nucleus (C)	1071	1.94E-03
regulation of meiosis (P)	12	2.00E-03
rRNA modification (P)	15	3.52E-03
diaminohydroxyphosphoribosylaminopyrimidine deaminase activity (F)	1	3.60E-03
DNA replication initiation (P)	24	3.83E-03
septin ring (C)	6	4.55E-03
nuclear mRNA splicing, via spliceosome (P)	72	4.68E-03
lagging strand elongation (P)	16	5.31E-03
glucosamine 6-phosphate N-acetyltransferase activity (F)	1	5.39E-03

Letter in parentheses indicates: P, Process; F, Function; C, Component. Number of associated genes indicates the number of genes that are assigned to a particular GO term.

Around 20 genes sharing the GO term "transcriptional regulator activity" also scored high. These genes may provide insight into the regulatory pathways that might be responsible for observed transcriptional response in this variety of processes. This set involves not only global transcriptional regulators such as Ume6p, but also specific regulators, especially related to cell cycle and DNA repair.

### Reporter TFs analysis

We used a network reconstruction depicting each known yeast transcription factor or regulatory protein connected to all genes known to being affected by these proteins. Using this graph we could identify Reporter Transcription Factors (or more correctly Reporter Regulators) indicating a significant collective change in the expression of the genes regulated by them (Table 4). For the complete list of reporter TFs see additional file 2. Reporter TFs highlight the regulatory pathways affected following a perturbation, and thus uncover the functional links between the perturbation and the regulatory mechanisms invoked in the cell. Since many TFs/regulators are regulated at post-transcriptional level, the expression of the reporter TFs/regulators themselves may or may not change *per se*. Nevertheless, identification of a particular protein as reporter-TF is a strong indication for the change in its activity following *SDH3 *deletion. The most marked reporter-TF emerging from this analysis is the Hap complex, for which the expression levels of Hap4 and Hap2 are also significantly changed. This confirms the association of Hap4 to the control of respiration [24]. Other reporter-TFs represent potential mediators of the observed global transcriptional changes as their involvement range from the control of metabolism (*GCR1, THI2*, and *SLN1*) to cell cycle (*FAR1, SWI6, YHP1 *and *YOX1*).

**Table 4:** Reporter-Transcription Factors for the *sdh3*Δ mutant

**Transcription Factor/Regulator**	**Number of associated genes**	**Reporter *p*-value**	**Expression *p*-value**	**Expression fold change**
HAP5	22	1.56E-03	0.262	1.68
HAP4	26	2.27E-03	0.037	-3.36
SLY1	12	3.33E-03	0.341	-1.71
HAP3	25	4.22E-03	0.186	-1.34
SLN1	2	5.71E-03	0.008	-1.91
TRS130	1	1.09E-02	0.015	-1.86
HAP2	28	1.39E-02	0.007	-2.16
FAR1	2	1.47E-02	0.310	-1.32
HYM1	2	1.94E-02	0.148	-1.55
THI2	8	2.30E-02	0.199	1.71
RAP1	26	2.45E-02	0.294	1.97
SWI6	31	2.92E-02	0.289	-1.48
YHP1	1	3.06E-02	0.020	-1.52
YOX1	1	3.06E-02	0.437	1.68
GCR1	18	3.45E-02	0.139	-2.15
THI3	9	3.60E-02	0.514	1.21
CMD1	1	3.87E-02	0.148	2.60

Number of associated genes denotes the genes that are regulated by the corresponding TF/Regulator as documented at the Yeast Proteome Database. Reporter *p*-value indicates the significance of the average change in the regulated genes. Note that there is no necessary correlation between the reporter *p*-value and *p*-value of the expression change in the level of the regulator itself.

### Promoter analysis

We searched for conserved sequence motifs in the upstream region of the genes with significant transcriptional response. With all the 434 genes queried together, no significantly over represented motif was found. However, when up and down regulated genes were queried separately we identified significantly over-represented sequence motifs. About 40% of up-regulated genes contain a 6-mer "TCGTCA" in their promoter region, while this motif is characteristic for only 20% of the genes in the whole genome. 29% of the significantly down-regulated genes also contain this motif in their promoter region. The list of all the significant genes with this motif is provided as supplementary information (additional file 3). The sequence TCGTCA corresponds to the known DNA binding site for the Autonomously Replicating Sequence (ARS) binding transcription factor Abf1p, a multifunctional global regulator [25]. Binding motifs for certain other transcriptional regulators such as Hsf1p (Heat shock transcription factor) and Ino4p (Inositol requiring) were also identified in the upstream regions of significantly down-regulated genes. Expression levels of *ABF1 *and *INO4 *were altered significantly in the *SDH3 *deletion mutant.

### Reporter Metabolite and subnetwork analysis

To gain further insight into the transcriptional response to the deletion of *SDH3*, we integrated the transcriptome data with the genome-scale yeast metabolic model [16] and thus identified so-called reporter metabolites [14] (methods). This method is based on the hypothesis that expression of genes encoding enzymes must be coordinated in order to satisfy the metabolic demands of the cell and corresponding stoichiometric constraints. The algorithm enables identification of so-called reporter metabolites (metabolites around which the most significant transcriptional changes occur) and enzyme sub-networks that respond significantly to genetic or environmental perturbations [14], in this case deletion of *SDH3*. Table 5 shows the key reporter metabolites (for the complete list see additional file 2) that were identified and it highlights metabolites involved in the TCA cycle (Isocitrate, Oxalosuccinate, FADH_2_), in the ergosterol biosynthesis pathway (zymosterol) and in phospholipid biosynthesis (ethanolamine).

**Table 5:** List of the top scoring reporter metabolites

**Metabolite**	**Number of neighbours**
Zymosterol	2
5-Amino-6-(5'-phosphoribosylamino)uracil	1
5-Amino-6-(5'-phosphoribitylamino)uracil	1
AMP	38
Isocitrate	5
L-Glutamyl-tRNA(Glu)	1
FADH2M	5
Ethanolamine	1
Aminoimidazolo ribotide	2
FADM	6
Oxalosuccinate	2
4-Amino-5-hydroxymethyl-2-methylpyrimidine	3
4-Amino-2-methyl-5-phosphomethylpyrimidine	3
2,5-Diamino-6-hydroxy-4-(5'-phosphoribosylamino)-pyrimidine	2
4,4-Dimethylzymosterol	2
NAD+M	17
2-Methyl-4-amino-5-hydroxymethylpyrimidine diphosphate	2
Uroporphyrinogen III	3
naxt	1
Sodium	1

Only the top-20 reporter metabolites are shown. Number of neighbours indicates the number of enzyme-coding genes whose products catalyze reactions involving that metabolite either as a substrate or as a product.

### Transcriptional readjustments in respiratory pathways

Since Sdhp provides reducing power to the respiratory chain, we expected that the *SDH3 *deletion would also lead to changes in genes involved in the respiratory pathways. Indeed, we observed that several genes involved in the oxidative phosphorylation, including, cytochrome c oxidase subunits/assembly (*COX 5B/6/7/8/10/12/13/14/15/18/20*), heme biosynthesis (*HEM3/14*), mitochondrial F_1_F_0 _ATP-synthase (*ATP18*), ubiquinone biosynthesis (*COQ3/5*, *CBP4*), and bc1p complex (*CYT1, RIP1, ABC1, COR1, QCR6/7*) were affected following the deletion. The majority of these genes were down regulated except for *COX14/15 *and *COQ3*, possibly due to different regulatory mechanisms governing their expression.

## Discussion

Results from the physiological characterization indicate that the *SDH3 *deletion mutant shows a growth defect under both aerobic and anaerobic conditions. This observation was partly expected for aerobic conditions and could be attributed to a major reduction in the respiratory capacity of the mutant strain, which is also supported by the carbon limited chemostat experiments. However, as the flux through the TCA cycle of the reference strain at the studied growth conditions is very low [26] due to glucose repression of respiration, it clearly points out that Sdh3 plays an additional role than being involved in respiration. This hypothesis is confirmed by our finding that there is a similar reduction in specific growth rate under anaerobic conditions. This additional role of Sdh3 may be different at the two different growth conditions. Thus, at anaerobic growth conditions it is likely that Sdh3 simply plays a role in redox balancing, whereas at aerobic growth conditions where there is predominantly fermentative metabolism, it may still be important to have functional respiration in order to prevent oxidative damage in the cells. Treatment with respiratory inhibitors antimycin and oligomycin had no effect on growth and final biomass yield of the *sdh3*Δ mutant while it severely affected biomass production of the reference strain. These additional experiments with mitochondrial inhibitors demonstrated that *SDH3 *deletion is sufficient to impair the electron transport system preventing the oxidation of NADH and succinate. However, the general down regulation of respiratory genes clearly implies that the cells are not attempting to restore their respiratory capacity. This is in line with the results obtained for respiratory deficient petite cells grown on non-repressive raffinose [27].

Off gas profiles during the batch experiments showed that the oxygen uptake rate in *sdh3*Δ is ~80% lower compared to the reference strain. Since oxygen is not used as final electron acceptor in the respiratory chain, as inferred from the experiments with mitochondrial inhibitors, the oxygen consumed is used in other cellular pathways. In yeast species non respiratory oxygen consumption is favoured when respiratory functions are impaired or absent, however, little is known about the non-respiratory oxygen uptake of aerobically growing cells.

During anaerobiosis, *S. cerevisiae *strongly increases glycerol production to provide for non-respiratory oxidation of NADH to NAD^+^, and consistent with the hypothesis of an altered redox metabolism originating from the deletion of *SDH3 *and consequent impairment of oxidative phosphorylation, a higher glycerol production was observed in aerobic cultivations of the mutant strain (similar yield as obtained in anaerobic cultivation). Thus, the deletion strain seems to readjust its metabolic fluxes mimicking growth under anaerobic conditions.

In an attempt to uncover the molecular mechanisms behind the observed global transcriptional response, we searched for significantly over-represented sequence motifs in the promoter regions of genes with significantly changed expression in the *SDH3 *mutant. This analysis revealed a binding site for Abf1p in 40% of the significantly up-regulated genes. Several different chromatin-related events such as DNA replication [28], gene silencing [29], chromatin remodeling [30], nucleotide excision repair [31], and gene activation and repression [25] involve direct binding of Abf1p. The genes that are reported to be transcriptionally regulated by Abf1p are involved in a multitude of cellular processes including carbon source regulation [32,33] nitrogen utilization [34,35] sporulation [36], meiosis [37], and ribosomal biogenesis [30,38]. Since these processes are also significantly affected in the *SDH3 *mutant, Abf1p appears to be one of the major mediators of the transcriptional response following the *SDH3 *disruption and consequent respiratory impairment. This is further supported by the fact that *ABF1 *transcription is significantly down regulated by more than four-fold in the *SDH3 *mutant, whereas under usual conditions *ABF1 *is found to be present in abundance [28,39]. Down regulation of *ABF1 *under anaerobic conditions also supports its role as a mediator of transcriptional changes in response to the absence of respiration. The binding motif recognized by Ino4p was also identified in the promoter analysis. Ino4p is a known regulator of genes involved in phospholipid synthesis [40,41], some of which also showed altered expression in the *SDH3 *mutant (*ACC1, CHO1, EKI1 and DPP1)*.

The reporter metabolites indicate specific parts of metabolism where significant transcriptional regulation is exerted, either to maintain homeostasis and/or to adjust the metabolite levels to altered demands. Two of the top scoring reporter metabolites suggested an interesting connection between mitochondrial impairment and lipid metabolism. Zymosterol is the immediate precursor of ergosterol, the major yeast sterol, which has a functional similarity to cholesterol in higher eukaryotic membranes, and is only synthesised in yeast under aerobic conditions. Our data show up-regulation of *AUS1*, an important sterol uptake gene normally expressed under anaerobic conditions, and down regulation of several key genes in the ergosterol biosynthesis pathway (*ERG11*, *ERG25*, *ERG27*, *ERG5*, *ERG9*) due to the deletion of *SDH3*. This information indicates the presence of a link that connects mitochondrial respiration and/or TCA cycle activity to the sterol biosynthetic pathways. We postulate that one of the mediators of this functional link is Hap1p which is known to positively regulate the *AUS1 *expression that is up-regulated in the *SDH3 *deletion mutant. Since the effect of mitochondrial impairment on lipid metabolism has been a focus of several studies related to metabolic diseases such as diabetes, our results may help in identifying similar regulatory mechanisms in human cells.

Ethanolamine is a precursor for the synthesis of phosphatydilethanolamine (PE), one of the major phospholipids components in cellular membranes of *S. cerevisiae*, and its role is crucial for growth when mitochondrial function is required [42]. In humans impairment or alterations in the phospholipids metabolism are suggested to represent important aspects of certain diseases [43]. Expression of several genes involved in PE biosynthesis such as *EKI1 *(ethanolamine kinase/choline kinase), *EPT1 (*dyacil-glycerol-ethanolamine-phosphotrasferase), and *CHO1 *(phosphatidylserine synthase) is known to be transcriptionally regulated by Ino4p, and they are all down-regulated in the *sdh3*Δ strain. As found in the promoter analysis, the binding motif of Ino4p was significantly over-represented in the promoter region of some of the significantly down-regulated genes, making it another potential mediator of the signal between respiratory chain and lipid metabolism.

**Figure 1:**
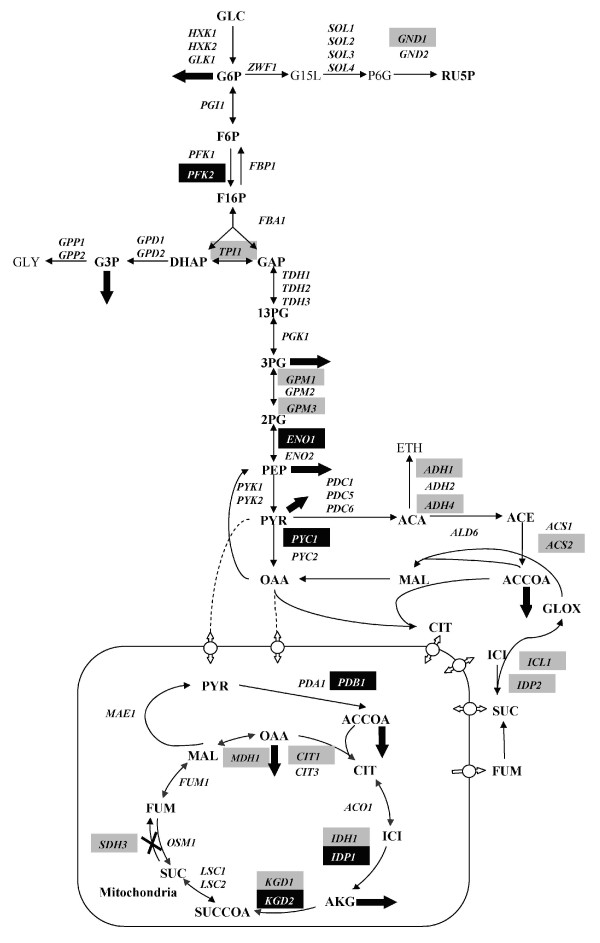
**Schematic representation of the up and down-regulated genes in the central carbon metabolism following *SDH3 *deletion**. Up-regulated genes are placed in black boxes, and down-regulated genes are placed in grey boxes. Thick arrows: drain of metabolites towards biomass production. Metabolites nomenclature: ACCOA, acetyl coenzyme A; ACA, acetaldehyde; ACE, acetate; AKG, 2-oxoglutarate; CIT, citrate; DHAP, dihydroxyacetone phosphate; ETH, ethanol; F6P, D-Fructose 6-phosphate; F16P D-Fructose 1,6-bisphosphate, FUM fumarate, GAP D-Glyceraldehyde 3-phosphate, G3P, Glycerol 3-phosphate; G6P, D-Glucose 6-phosphate; G15L, D-Glucono-1,5-lactone 6-phosphate; GLY, Glycerol; GLOX, Glyoxylate; GLC, Glucose; ICI, Isocitrate; MAL, Malate; OAA, Oxaloacetate; P13G, 3-Phospho-D-glyceroyl phosphate; P2G, 2-Phospho-D-glycerate; P3G, 3-Phospho-D-glycerate; P6G, 6-Phospho-D-gluconate; PEP, Phosphoenolpyruvate; PYR, pyruvate; RU5P, D-Ribulose 5-phosphate; SUC, Succinate; SUCCOA, Succinyl CoA.

Certain other metabolites including isocitrate, oxalosuccinate, and FADH_2 _involved in the TCA cycle also emerge as reporter metabolites, and together with the altered expression of many genes related to the TCA and glyoxylate cycles (Figure 1) they possibly suggest alterations of Acetyl-CoA metabolism (see additional file 4 for supporting information).

**Figure 2:**
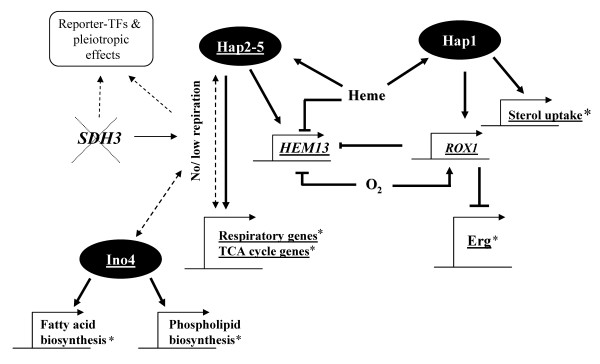
**Proposed regulatory model of the effects of *SDH3 *deletion**. The model indicates a regulatory link that connects the respiratory dysfunction resulting from *SDH3 *deletion to changes in fatty acid metabolism and other pathways. Deletion of *SDH3 *leads to no/low respiration which is hypothesized to trigger regulatory circuits involving Hap2/3/4/5 complex, Hap1 and Ino4. Links that we could not find any data/literature evidences about are denoted by broken lines. Underlined gene names indicate down-regulation, while * denotes the genes/pathways for which similar changes in expression have been observed under anaerobic conditions for the reference strain.

In accordance with our results, transcription profiling of a collection of mutants defective in each of the TCA cycle genes also showed transcriptional changes in a large number of genes, *albeit *under non-repressive conditions [44]. Taken together, our results suggest that nuclear gene signalling is responsive to TCA cycle dysfunction. Many of the transcriptional changes following the deletion of *SDH3 *are located in fatty acid and sterol metabolism. What is the regulatory link that connects the respiratory dysfunction to changes in fatty acid metabolism and other pathways? Towards investigating this question, inferences and postulates based on our analysis are summarized in the qualitative regulatory model represented in Figure 2. In *S. cerevisiae *the heme-activated complex Hap2/3/4/5 is known to regulate the expression of many of the TCA cycle and respiratory chain genes, although neither the heme nor the oxygen regulation of the Hap-complex is clearly understood. The Hap-complex appears as one of the transcription factors with altered expression level in the *SDH3 *mutant. Moreover several of the genes regulated by the Hap-complex were also responsive to this change as indicated by Reporter-TF analysis. Thus, it may be hypothesized that the deletion of the *SDH3 *gene (and perhaps respiratory deficiency in general) might be in the first place responsible for causing the down-regulation of genes coding for the Hap-complex, which in turn would explain the down-regulation of respiratory genes and some of the TCA cycle genes. The Hap-complex has also been found to down-regulate the expression of heme biosynthesis pathway [24]. Consequently, the up-regulation of *HAP1*, another heme-responsive transcription factor, can be attributed to reduced heme biosynthesis. Hap1p is involved in the regulation of several pathways including sterol biosynthesis and uptake, and is essential during anaerobic or heme-deficient growth. *AUS1 *(coding for sterol uptake protein) and several genes involved in the ergosterol, fatty acid and phospholipid biosynthesis show differential gene expression in the *SDH3 *mutant that is consistent with the changes observed in the reference strain under anaerobic conditions [45]. Our analysis suggests that Ino4p may play a key role in the regulation of these genes following *SDH3 *deletion, which is consistent with the known regulatory targets of Ino4p. However, it is unclear how the Ino4p regulation is triggered.

## Conclusion

Overall, we present a genome-wide transcription analysis of *SDH3 *deleted *S. cerevisiae *mutant grown under glucose-repressed conditions. By using an integrative systems biology approach we reconstructed a qualitative regulatory map that links mitochondrial respiration with fatty acid and sterol metabolism. Several bioinformatics and systems biology algorithms were employed to elucidate the transcriptional hot-spots in different cellular networks, including metabolic networks and transcriptional regulatory networks that significantly respond to the deletion of *SDH3*. Several regulatory proteins including the Hap-complex, Hap1, Ino4, Abf1 and Sln1 appear to play an important role in mediating the cellular response following respiratory defect at the succinate dehydrogenase node.

## Authors' contributions

DC constructed the strain, performed cultivation and microarray experiments, drafted the manuscript and participated in design and data analysis. KRP assisted in cultivation experiments, drafted the manuscript, helped in data analysis and designed the study. CS participated in the design of the study. JN conceived, designed and coordinated the study. All authors read, helped in drafting, and approved the final manuscript.

## Supplementary Material

Additional File 1 **Data from microarray analysis of the sdh3Δ and reference strain.** Results obtained from the t-test and q-test. Click here for file

Additional File 2 **Reporter features.** Complete list of reporter TFs, reporter GOs and reporter metabolites. Click here for file

Additional File 3 **Promoter analysis.** List of all the significantly changed genes that have Abf1 binding motif in their promoter region. Click here for file

Additional File 4 **Supporting information.** Detailed discussion on phenotypic effects observed in the *sdh3*Δ mutant and reporter metabolites analysis. Click here for file
