# Supplementary material for: Global transcriptional response of Saccharomyces cerevisiae to the deletion of SDH3
Source: BMC Syst Biol. 2009 Feb 6;3:17. doi: 10.1186/1752-0509-3-17 (PMC2661886; doi:10.1186/1752-0509-3-17)
Supplement: Additional File 4 — Supporting information. Detailed discussion on phenotypic effects observed in the sdh3Δ mutant and reporter metabolites analysis. [file 1752-0509-3-17-S4.pdf]

Detailed discussion on phenotypic effects observed in the *sdh3*Δ mutant and reporter metabolites analysis.

(Global transcriptional response of *Saccharomyces cerevisiae* following the deletion of succinate dehydrogenase. Donatella Cimini, Kiran Raosaheb Patil, Chiara Schiraldi and Jens Nielsen)

Two major and interconnected biochemical conversions occur in the mitochondria: oxidative phosphorylation, responsible for synthesising ATP and oxidising-reducing equivalents, and the TCA cycle, responsible for supplying precursors for biomass production. Direct inhibition of the respiratory chain or the disruption of the Sdhp complex leads to a redirection of metabolic carbon flux from the TCA cycle to alternative routes necessary for growth and reoxidisation of NAD(P)H. Among the phenotypic effects observed in the *sdh3*Δ mutant is the increased extra-cellular secretion of pyruvate (Table 1). Genes involved in pyruvate utilization were also up-regulated (*PYC1*, *PDB1*), suggesting that intracellular pyruvate concentration was increased due to the lack of TCA cycle activity. In genome-wide transcription analysis of petite mutants up-regulation of *PYC1* and *ACS1* was observed which resulted in increased pools of oxaloacetate and acetyl CoA [1]. Intracellular increases in the pyruvate pool of the *sdh3*Δ mutant may help increasing the production of ethanol and glycerol, as observed in non-oxidative cells for regeneration of NAD^+. However, expression of *GPD2* (glycerol-3-phosphate dehydrogenase) was not changed in the *SDH3* mutant in contrast to the observations in other studies with respiratory deficient cells [1]. This difference may be attributed either to glucose repression [2], and/or to the high concentration of accumulated NADH that may be sufficient to drive the reaction towards NADH oxidation without necessity for transcriptional up-regulation of *GPD2*. Altered glycerol production may also be attributed to the reporter-TF *SLN1,* of which the transcription level was also significantly down-regulated in the mutant.

The reporter metabolites indicate specific parts of metabolism where significant transcriptional regulation is exerted, either to maintain homeostasis and/or to adjust the metabolite levels to altered demands. Zymosterol was identified as the top scoring reporter metabolite following the *SDH3* deletion and is the immediate precursor of ergosterol, the major yeast sterol which has a functional similarity to cholesterol in higher eukaryotic membranes. In fact, synthesis, regulation, and esterification of zymosterol and ergosterol are comparable in yeast and human [3]. Membrane lipids in eukaryotes play essential biological functions spanning from membrane trafficking to signal transduction [4]. It is therefore interesting to investigate the effect of mitochondrial impairment on lipid metabolism which has also been a focus of several studies related to metabolic diseases such as diabetes. The sterol biosynthesis pathway in *S. cerevisiae* represents a complex oxygen-dependent pathway, whereby most of the *ERG* genes are regulated by oxygen [5]. Still unidentified molecular mechanisms are used by endogenous and exogenous sterols to regulate gene expression. It has been suggested that sterol requirement for growth and/or the presence of other lipids could also be responsible for the regulation of these pathways [6]. Under anaerobic growth conditions yeast cannot synthesize aromatic sterols, and exogenously supplied sterols are required. However, in the presence of oxygen the expression of sterol uptake genes is usually down-regulated and yeast does not take up sterols, this phenomenon is often referred to as “aerobic sterol exclusion” [7]. Conversely, an important sterol uptake gene *AUS1* (a member of the ATP-binding cassette (ABC) superfamily of membrane transporters) [8] was significantly up-regulated in the *SDH3* deleted strain under aerobic conditions. Yeast cells are known to bypass the “aerobic sterol exclusion” under aerobic growth conditions only if they possess a hypermorphic allele of the transcription factor Upc2p or if they over-express the transcriptional regulator Sut1p [9]. Also cells that can not synthesize heme need to exogenously take up sterols during aerobic growth [10]. It is also reported that sterol uptake is not affected by the presence of mutations in the mitochondrial genome. Thus up-regulation of *AUS1* and down regulation of several key genes in the ergosterol biosynthesis pathway (*ERG11*, *ERG25*, *ERG27*, *ERG5*, *ERG9*) due to the deletion of *SDH3* implies the presence

of a link that connects mitochondrial respiration and/or TCA cycle activity to the sterol biosynthetic pathways. Our data suggest that one of the mediators of this functional link is Hap1p which is known to positively regulate the *AUS1* expression that is up-regulated in the *SDH3* deletion mutant.

Another high scoring reporter metabolite identified is ethanolamine which is the precursor for the synthesis of the phospholipid phosphatidylethanolamine (PE). PE functions in physiological processes such as cell signalling, membrane fusion, cell division, and apoptosis in both eukaryotic and prokaryotic cells [11,12]. It is one of the major phospholipids components in cellular membranes of *S. cerevisiae,* and its role is crucial for the growth when mitochondrial function is required [13]. In humans, the production of specialised PE, such as plasmalogen, is severely impaired in peroxisomal disorders such as the Zellweger syndrome [14]. Furthermore, it has recently been suggested that alterations in the phospholipid metabolism and mitochondrial abnormalities could represent an important aspect of Alzheimer's disease [15,16]. E.g. *CKI1* which codes for choline kinase and has a high homology and overlapping function to the yeast *EKI1* gene is down-regulated in Alzheimer's disease and is over-expressed in several cancer cells [17]. Deletion of *SDH3* in *S. cerevisiae* also leads to the down-regulation of the *EKI1* gene, suggesting an interesting parallel. Expression of several genes involved in PE biosynthesis such as *EKI1* (ethanolamine kinase/ choline kinase), *EPT1 (*dyacil-glycerol-ethanolamine-phosphotrasferase), and *CHO1* (phosphatidylserine synthase) is known to be transcriptionally regulated by Ino4p, and they are all down-regulated in the *sdh3*Δ strain. As mentioned in the promoter analysis section, the binding motif of Ino4p was significantly over-represented in the promoter region of some of the significantly down-regulated genes, making it another potential mediator of the signal between respiratory chain and lipid metabolism.

Certain other metabolites including isocitrate, oxalosuccinate, and $FADH_2$ involved in the TCA cycle also emerge as reporter metabolites. Furthermore, as depicted in Figure 1, the expression of many related genes such as *CIT1*, *MDH1*, *KGD1*, *KGD2*, *ICL1*, *IDP2*, *ACS2*, and *ALD5,* that link

the TCA cycle with the glyoxylate cycle and fatty acid biosynthesis have altered expression in the *SDH3* mutant. Our hypothesis is that as a consequence of the increased fluxes towards ethanol, the expression of genes responsible for Acetyl-CoA synthesis and utilization may be down-regulated (*ALD5, ACS2, IDP2, ICL1*) as a result of its decreased availability. *ACC1*, which codes for Acetyl-CoA carboxylase and is responsible for the first and committed step in fatty acid biosynthesis, is down-regulated in the *SDH3* mutant. Down-regulation of this gene may also be due to the absence of Acetyl-CoA necessary to drive the reaction. Alternatively, decreased expression of *ACC1* can be attributed to the transcriptional activator Ino4p. *ACC1* has 46 % identity with the human Acetyl CoA carboxylase-beta that is reported to be down-regulated in type II diabetes mellitus patients and in obese subjects following weight loss [18,19].

References

1. Epstein CB, Waddle JA, Hale W, Dave V, Thornton J, Macatee TL, Garner HR, Butow RA: **Genome-wide responses to mitochondrial dysfunction.** *Mol Biol Cell* 2001, **12:** 297-308.

2. Nevoigt E, Stahl U: **Reduced pyruvate decarboxylase and increased glycerol-3-phosphate dehydrogenase [NAD+] levels enhance glycerol production in Saccharomyces cerevisiae.** *Yeast* 1996, **12:** 1331-1337.

3. RINE JASP: **The Yeast Saccharomyces cerevisiae in Molecular and Cellular Biology: A Smaller but not Lower Eucaryote.** *Amer Zool* 1989, **29:** 605-616.

4. Daum G, Lees ND, Bard M, Dickson R: **Biochemistry, cell biology and molecular biology of lipids of Saccharomyces cerevisiae.** *Yeast* 1998, **14:** 1471-1510.

5. Sturley SL: **Conservation of eukaryotic sterol homeostasis: new insights from studies in budding yeast.** *Biochim Biophys Acta* 2000, **1529:** 155-163.

6. Rosenfeld E, Beauvoit B: **Role of the non-respiratory pathways in the utilization of molecular oxygen by Saccharomyces cerevisiae.** *Yeast* 2003, **20:** 1115-1144.

7. Lewis TA, Taylor FR, Parks LW: **Involvement of heme biosynthesis in control of sterol uptake by Saccharomyces cerevisiae.** *J Bacteriol* 1985, **163:** 199-207.

8. Wilcox LJ, Balderes DA, Wharton B, Tinkelenberg AH, Rao G, Sturley SL: **Transcriptional profiling identifies two members of the ATP-binding cassette transporter superfamily required for sterol uptake in yeast.** *J Biol Chem* 2002, **277:** 32466-32472.

9. Crowley JH, Leak FW, Jr., Shianna KV, Tove S, Parks LW: **A mutation in a purported regulatory gene affects control of sterol uptake in Saccharomyces cerevisiae.** *J Bacteriol* 1998, **180:** 4177-4183.

10. Raychaudhuri S, Prinz WA: **Uptake and trafficking of exogenous sterols in Saccharomyces cerevisiae.** *Biochem Soc Trans* 2006, **34:** 359-362.

11. Carman GM, Zeimetz GM: **Regulation of phospholipid biosynthesis in the yeast Saccharomyces cerevisiae.** *J Biol Chem* 1996, **271:** 13293-13296.

12. Henry SA, Patton-Vogt JL: **Genetic regulation of phospholipid metabolism: yeast as a model eukaryote.** *Prog Nucleic Acid Res Mol Biol* 1998, **61:133-79.:** 133-179.

13. Storey MK, Clay KL, Kutateladze T, Murphy RC, Overduin M, Voelker DR: **Phosphatidylethanolamine has an essential role in Saccharomyces cerevisiae that is independent of its ability to form hexagonal phase structures.** *J Biol Chem* 2001, **276:** 48539-48548.

14. Farooqui AA, Rapoport SI, Horrocks LA: **Membrane phospholipid alterations in Alzheimer's disease: deficiency of ethanolamine plasmalogens.** *Neurochem Res* 1997, **22:** 523-527.

15. Ellison DW, Beal MF, Martin JB: **Phosphoethanolamine and ethanolamine are decreased in Alzheimer's disease and Huntington's disease.** *Brain Res* 1987, **417:** 389-392.

16. Bonilla E, Tanji K, Hirano M, Vu TH, DiMauro S, Schon EA: **Mitochondrial involvement in Alzheimer's disease.** *Biochim Biophys Acta* 1999, **1410:** 171-182.

17. de Molina AR, Gallego-Ortega D, Sarmentero J, Banez-Coronel M, Martin-Cantalejo Y, Lacal JC: **Choline kinase is a novel oncogene that potentiates RhoA-induced carcinogenesis.** *Cancer Research* 2005, **65:** 5647-5653.

18. Musi N, Hirshman MF, Nygren J, Svanfeldt M, Bavenholm P, Rooyackers O, Zhou G, Williamson JM, Ljunqvist O, Efendic S, Moller DE, Thorell A, Goodyear LJ: **Metformin Increases AMP-Activated Protein Kinase Activity in Skeletal Muscle of Subjects With Type 2 Diabetes.** *Diabetes* 2002, **51:** 2074-2081.

19. Rosa G, Manco M, Vega N, Greco AV, Castagneto M, Vidal H, Mingrone G: **Decreased Muscle Acetyl-Coenzyme A Carboxylase 2 mRNA and Insulin Resistance in Formerly Obese Subjects.** *Obesity Res* 2003, **11:** 1306-1312.
